# A Systematic Review of Apoptosis in Correlation With Cancer: Should Apoptosis Be the Ultimate Target for Cancer Treatment?

**DOI:** 10.7759/cureus.28496

**Published:** 2022-08-28

**Authors:** Abdelrahman Abaza, Advait M Vasavada, Akhil Sadhu, Carla Valencia, Hameeda Fatima, Ijeoma Nwankwo, Mahvish Anam, Shrinkhala Maharjan, Zainab Amjad, Safeera Khan

**Affiliations:** 1 Pathology, California Institute of Behavioral Neurosciences & Psychology, Fairfield, USA; 2 Internal Medicine, California Institute of Behavioral Neurosciences & Psychology, Fairfield, USA; 3 Family Medicine, California Institute of Behavioral Neurosciences & Psychology, Fairfield, USA; 4 Research, California Institute of Behavioral Neurosciences & Psychology, Fairfield, USA

**Keywords:** multiple drug resistance, anti-apoptotic therapy, cancer, regulated cell death, apoptosis

## Abstract

Targeting apoptosis in cancer therapy has become increasingly popular, and there has been an increasing debate on whether apoptosis should be one of the main targets of therapy in cancer management. This study demonstrates the definition of apoptosis, the signaling pathways, and the pathogenesis behind it. We also show the correlation between apoptosis and cancer and how cancer can evade apoptosis to develop resistance to therapy. In addition, we illustrate the efficacy of adding pro-apoptotic therapy to conventional radio-chemotherapy cancer treatment.

A systematic review was conducted using PubMed, PubMed Central (PMC), and ResearchGate, including papers written in English, focusing on adult and geriatric populations, in literature reviews, systematic reviews, and randomized controlled trials published in the last 25 years with relevance to the question.

Based on the findings of this review, we conclude that apoptosis is a very sophisticated programmed cellular death with many signaling pathways. Its evasion should be considered one of the hallmarks of cancer and is responsible for multiple drug resistance (MDR) to cancer therapy. Targeting apoptosis seems promising, especially if combined with radio-chemotherapy.

## Introduction and background

The pathways of cell death were first recognized by the work of Robert Horvitz while studying cell fate in lower organisms such as Caenorhabditis elegans, which eventually helped him to win the 2002 Nobel Prize in Physiology or Medicine. Much was learned about the cell death mechanisms from within a cell and through the immune system [1]. One of the most important ones was apoptosis, a form of programmed cell death that leads to the efficient removal of damaged cells, such as those occurring during development or after deoxyribonucleic acid (DNA) damage [2]. An important feature of apoptosis is that it performs its actions fundamentally through a subtype of serine proteases called caspases, which are cysteinyl proteases that proteolytically cleave different nuclear as well as cytoplasmic constituents. These caspases comprise 11 members, grouped into three main groups, of which groups two (caspases 2, 3, 7) and three (caspases 6, 8, 9, 10) are involved in apoptosis. Caspases are eventually responsible for the destruction of cells depending on various signaling pathways [3]. The apoptosis pathogenesis is complex and involves two main signaling pathways: extrinsic and intrinsic. Both activate the effector apoptotic caspases, eventually resulting in morphological and biochemical cellular alterations characteristic of apoptosis [4,5]. One of the most important determinants of whether the cell will undergo apoptosis or not is the balance between the pro-apoptotic and anti-apoptotic protein regulators. In precancerous lesions, DNA damage can induce apoptosis to remove potentially harmful cells, blocking tumor growth. In contrast, the disorganization of apoptosis can lead to unchecked cellular proliferation, cancer development, and cancer resistance to drug therapies [6]. Cancer cells often over-express different proteins that have a major contribution in resisting the cascade of apoptosis. Multiple mechanisms induced by cancer cells rescue them from programmed cell death, especially through the over-expression of the anti-apoptotic molecules [7]. In fact, the majority of apoptosis signaling research is dependent on B-cell lymphoma 2 homology 3 (BH3) proteins [8]. There is an equilibrium between pro-survival and pro-death BH3 proteins. When that equilibrium sways toward pro-death BH3 proteins, apoptosis tends to happen, but when it sways toward pro-survival proteins, this leads to the activation of survival signaling, which leads to pathological conditions such as cancers.

With these remarkable findings, the discovery of new drugs accelerated to create small molecule inhibitors (SMI) that can target apoptosis pathway proteins like B-cell lymphoma 2 (Bcl-2), induced myeloid leukemia cell differentiation protein (Mcl-1), B-cell lymphoma extra-large (Bcl-xL), Bcl-2 related protein A1 (A1/Bfl1) and Bcl-2-like-protein-2 (Bcl2l2/Bcl-w). Despite that, some trends were proven to be of minimal success, and in different cases, the malignant cells tend to be unresponding to these apoptosis-prompting drugs [9]. However, progress over the last two decades has been made in cancer-targeted therapies through the blocking of various kinases that increase tumor progression, in part through cell proliferation and survival; insights have been emerging regarding the connections between the cell death mechanisms and their contribution to efficacy of cancer targeted therapies. Ultimately, tumor cell death is impacted by the balance between these pathways as well as the extrinsic immune anti-tumor mechanisms [10].

## Review

Methods

Preferred Reporting Items for Systematic Reviews and Meta-Analyses (PRISMA) guidelines were implemented [11]. That helped us a lot to achieve a smooth and systematic review experience.

Search Strategy

Worldwide databases, including PubMed, PubMed Central (PMC), and ResearchGate were selected to look for relevant information applying the Medical Subject Headings (MeSH) strategy [12-14].

The final MeSH strategy for PubMed, PMC is as follows: ((( "Apoptosis/drug effects"[Mesh] OR "Apoptosis/etiology"[Mesh] OR "Apoptosis/immunology"[Mesh] OR "Apoptosis/physiology"[Mesh] )) OR ( "Regulated Cell Death/drug effects"[Mesh] OR "Regulated Cell Death/immunology"[Mesh] OR "Regulated Cell Death/physiology"[Mesh] )) AND Apoptosis AND Cancer.

The keywords used for the ResearchGate database are "apoptosis" AND "cancer".

Inclusion and Exclusion Criteria

Inclusion and Exclusion criteria are shown in Table 1.

**Table 1:** Inclusion and exclusion criteria

Inclusion criteria	Exclusion criteria
1. Papers focusing on adult and geriatric population	1. Papers discussing pediatric population
2. Published literature reviews, systematic reviews, and randomized controlled trials	2. Case studies, observational studies, and grey literature
3. Papers selected from the previous 25 years	3. Papers published over 25 years ago
4. Intrinsic/extrinsic pathways of apoptosis	4. Necrosis, reversible cell injury
5. Papers written in the English language	5. Papers not written in the English language
6. Papers relevant to the question	6. Papers irrelevant to the question

Analysis of Study Quality

We thoroughly evaluated twelve selected studies using standardized quality assessment tools, ten of which were of medium or high quality. The following tools were used: Assessment of Multiple Systematic Reviews (AMSTAR) tool for the systematic review article, Scale for the Assessment of Narrative Review Articles (SANRA) checklist for traditional review articles, and Cochrane risk-of-bias assessment tool for Randomized Controlled Trials (RCTs).

One systematic review showing the relationship between B-cell lymphoma 2 (Bcl-2) and regulation of apoptosis after traumatic brain injury was assessed by AMSTAR criteria, as shown in Table 2.

**Table 2:** Assessment of Multiple Systematic Reviews (AMSTAR) criteria for systematic reviews

AMSTAR criteria (yes, partial yes, no)	Study
	Deng et al. [15]
Did the research questions and inclusion criteria for the review include patient/problem, intervention, comparison, and outcome (PICO) components?	Yes
Did the report of the review contain an explicit statement that pointed to the fact of the establishment of the methods before conducting the review?	Yes
Did the review authors explain their selection of the study designs for inclusion in the review?	Yes
Did the review authors use a comprehensive literature search strategy?	Yes
Did the review authors perform study selection in duplicate?	Yes
Did the review authors perform data extraction in duplicate?	Partial yes
Did the review authors provide a list of excluded studies and justify the exclusions?	Yes
Did the review authors describe the included studies in adequate detail?	Partial yes
Did the review authors use a satisfactory technique for assessing the risk of bias in the included studies?	Yes
Did the review authors report on the funding sources for the studies included in the review?	Yes
If a meta-analysis was performed, did the review authors use appropriate methods for the statistical combination of results?	Partial yes
If a meta-analysis was performed, did the review authors estimate the influence of risk of bias in one study over the meta-analysis findings?	Partial yes
Did the review authors account for the risk of bias in individual studies when interpreting/discussing the results of the review?	Yes
Did the review authors provide a satisfactory explanation for any kind of inconsistency seen in the results section?	Yes
Did the review authors report any potential sources of conflict of interest in order to conduct this review?	No
Total score	12/15

Seven review articles were assessed by SANRA quality assessment, as shown in Table 3, to demonstrate the in-depth quality of the presented review articles.

**Table 3:** Scale for the Assessment of Narrative Review Articles (SANRA) quality assessment for review articles

Publication	Neophytou et al. [16]	Ramzi et al. [17]	Carneiro et al. [18]	Xu et al. [19]	Strasser et al. [20]	Singh et al. [21]	Yuan et al. [22]
Justification of the article's importance in the readership	2	2	2	2	2	2	2
Statement of concrete aims or formulation of questions	2	1	1	2	1	2	2
Description of the literature search	2	2	2	2	2	2	2
Referencing	2	2	2	2	2	2	2
Scientific reasoning	2	2	1	2	2	2	2
Appropriate presentation of data	1	2	2	2	2	2	2

Two RCTs were assessed by Cochrane risk-of-bias, as shown in Table 4, to demonstrate the effects of targeting apoptosis in cancer treatment.

**Table 4:** Cochrane risk-of-bias for assessment of randomized controlled trials (RCTs)

RCT	Selection bias	Reporting bias	Performance bias	Detection bias	Attrition bias
Spanheimer et al. [23]	Low risk	Low risk	Low risk	Intermediate risk	Low risk
Zerp et al. [24]	Low risk	Low risk	Intermediate risk	Low risk	Low risk

Data Extraction

The papers acquired from databases were carefully chosen through multiple eligibility phases. The titles of promising papers were selected, and irrelevant ones were precluded with the removal of duplicate records. Afterward, the papers were tested for quality assessment, and those with any deficiencies were excluded.

**Figure 1:**
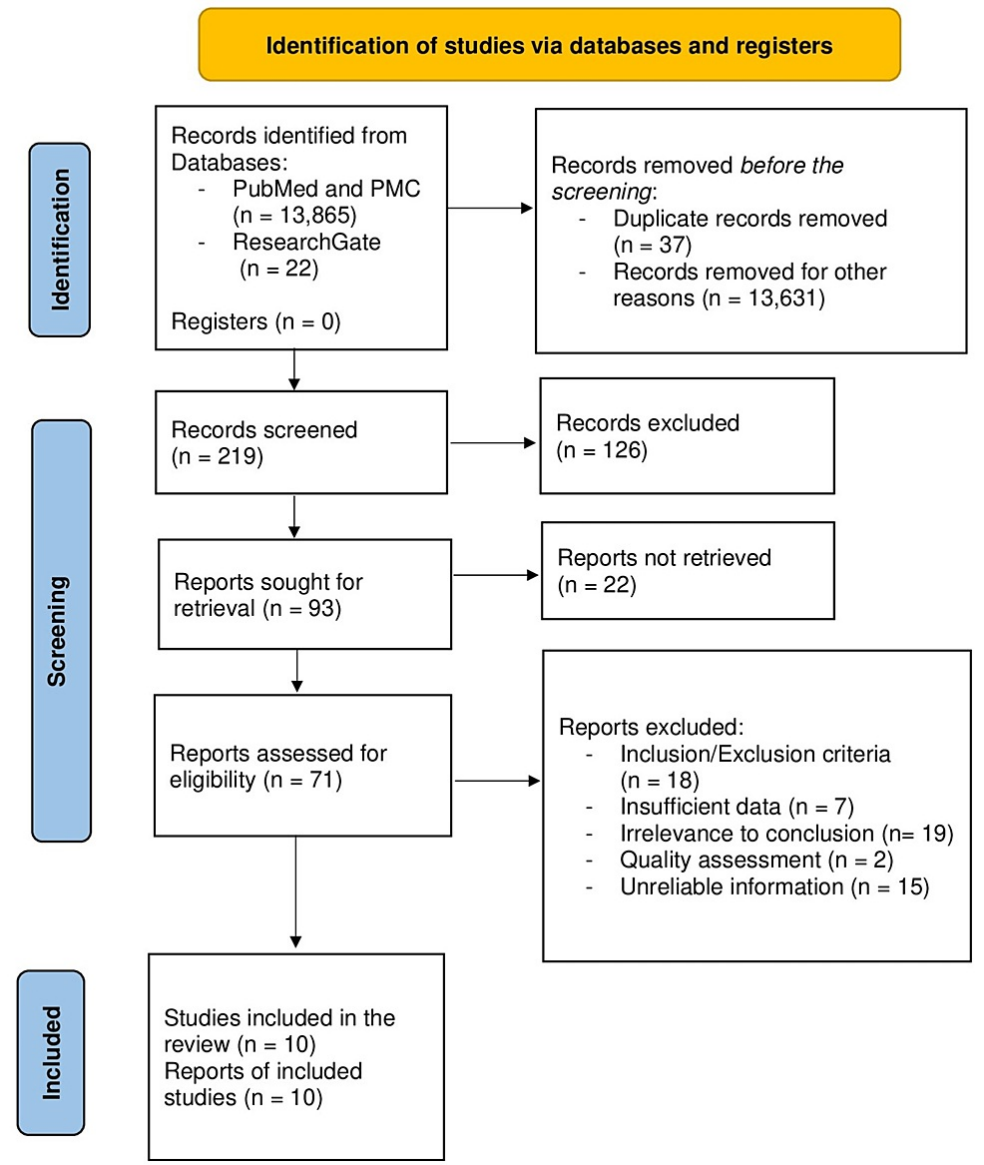
Data extraction process according to Preferred Reporting Items for Systematic Reviews and Meta-Analyses (PRISMA) flowchart

Relevant information was summarized in the following Figure 1 to show an overview of the data extraction process.

Results

Overall, 13,887 papers were established in the first search approach of databases. Of these papers, 37 got excluded due to duplicate records, and 13,631 were eliminated due to irrelevance to the topic being discussed. The researchers then read through the rest of the papers (n=219) to screen for the appropriate information; resulting in 71 research papers that were assessed for eligibility based on exclusion/inclusion criteria, relevance to the conclusion, data sufficiency, quality assessment, and reliability of information presented with ten research papers fulfilling all requirements to be included in our research paper. Of the ten included research papers, there were seven review articles, one systematic review, and two RCTs. Each article included was read and scrutinized.

Discussion

Apoptosis is a physiological programmed cell death that depends on extrinsic and intrinsic pathways that both lead to the enzymatic cleavage of cellular proteins through the action of effector caspases [25]. Apoptosis can also be caused by granzymes and perforins within cytotoxic granules in T-lymphocytes or natural killer (NK) cells [26]. The apoptosis process involves cellular breakdown with resulting cell shrinkage, chromatin condensation, membrane blebbing, and apoptotic body creation [27]. The nucleus can undergo pyknosis, karyorrhexis, and karyolysis with the cytoplasm turning deeply eosinophilic but cell membranes remain typically intact without inflammation, unlike necrosis. The breakdown of deoxyribonucleic acid (DNA) into nucleosomes was seen as DNA laddering on agarose gels [28].

Pathways for Apoptosis

There are two main pathways for apoptosis: the intrinsic and the extrinsic pathway.

1. The intrinsic pathway: The intrinsic pathway is the final common pathway for intrinsic apoptotic cell death, which involves mitochondrial outer membrane permeabilization (MOMP), with the release of cytochrome c from the mitochondria [29-31]. Bcl-2-associated X protein (BAX) and Bcl-2 antagonist killer 1 (BAK) form pores in the mitochondrial membrane resulting in cytochrome c release leading to activation of effector caspases that fragment the nucleus and degrade cellular cytoskeletal elements forming cytoplasmic and membrane blebs, which eventually form apoptotic bodies. It occurs after exposure to harmful stimuli such as DNA damage by radiation, toxins, and hypoxia or when a regulation factor is withdrawn from proliferating cells, such as the decrease in interleukin-2 (IL-2) following a completed immunological reaction causing apoptosis of proliferating effector cells. This pathway is regulated by Bcl-2 proteins, which include BAX, BAK, and BAD (Bcl-2 associated agonist of cell death) that positively regulate apoptosis and are considered "pro-apoptotic factors" in addition to Bcl-2, Bcl-xL, and Mcl-1 that negatively regulate apoptosis and are considered "anti-apoptotic factors". At the same time, Bcl-2 keeps the mitochondrial membrane impermeable, thereby preventing cytochrome c release. Therefore, in follicular non-Hodgkin lymphoma with Bcl-2 overexpression, there is a decrease in activation of caspases with a resultant decrease in cellular apoptosis and an increase in tumorigenesis. In addition, regulation of Bcl-2 proteins transcription can be achieved through the p53 tumor suppressor gene and cyclin-dependent kinase (CDK) [32]. Following cytochrome c liberation from the cellular mitochondria, activating caspases can also be successfully suppressed by inhibitors of apoptosis (IAP) proteins [33]. Therefore, the balance of the family of pro-apoptotic proteins versus the anti-apoptotic family members has always been considered a cellular rheostat that controls cell death in mammalian cells [34].

**Figure 2:**
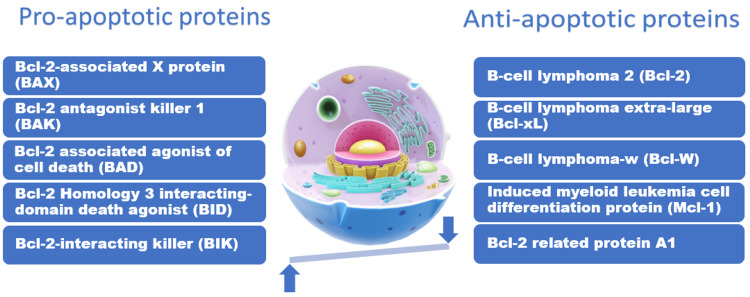
The balance between significant pro-apoptotic and anti-apoptotic proteins involved in the regulation of apoptosis Source: [35]

The balance between significant pro-apoptotic and anti-apoptotic proteins involved in the regulation of apoptosis can be illustrated in Figure 2.

2. The extrinsic pathway: A second common pathway of cell death, extrinsic cell death, can start apoptosis from cell membrane proteins known as death receptors. It depends on ligand-receptor interactions such as tumor necrosis factor-alpha (TNF-α) binding to TNF-α receptor and Fas ligand (FasL) binding to Fas receptors, which act as a death signal for the cells required to undergo apoptosis via caspases [36-38]. An illustrative example of the importance of Fas-FasL interaction is the negative selection of T lymphocytes in the thymic medulla that prevents the formation of self-reacting T cells capable of attacking the body's tissues. In addition, the p53 tumor suppressor transcriptionally upregulates Fas; therefore, drugs that activate p53 action can cause cell death depending on both intrinsic and extrinsic cell death pathways [39]. Another valuable extrinsic apoptotic pathway is the TNF-related apoptosis-inducing ligand (TRAIL) pathway. TRAIL was first recognized by its sequence homology to FasL [40]. An intrinsic protease cleaves TRAIL, releasing a ligand that can bind to cell surface receptors resulting in the triggering of an intracellular signaling cascade similar to the Fas pathway [41]. A third extrinsic apoptotic pathway depends on the release of intracytoplasmic granules by immune cells, such as the release of perforin and granzyme B by the cluster of differentiation 8 (CD8) positive cytotoxic T lymphocytes, which also activate effector caspases and therefore upregulates the apoptosis process.

Macrophages remove most cells undergoing apoptosis by packing remnants into vesicles known as apoptotic bodies (ApoBDs). Macrophages first respond to "find-me" signals which are released by cells undergoing apoptosis, followed by "eat-me" signals from the ApoBDs to phagocytose them, then cytoskeletal rearrangements and modifications of the phagocytes occur to enable the ingestion of ApoBDs [42,43].

Apoptosis in Correlation With Cancer and Development of Multiple Drug Resistance (MDR) to Apoptosis

Naturally, permanent DNA damage triggers cells to undergo apoptosis; for instance, the p53 gene can upregulate the expression of apoptosis-initiating proteins. In addition, it is capable of confronting the Bcl-2 and Bcl-xL anti-apoptosis functions. Therefore, cells incapable of undergoing apoptosis, like the ones with p53 mutations or increased expression of Bcl-2, may build up lots of mutations that promote the initiation of the malignancy process. Mutations of the p53 tumor suppressor would not only stop the cells from undergoing apoptosis, but in fact, they would also decrease some tumor suppression characteristics, such as the capability of activating the pathways of repair of DNA and the interception of the cell growth signals [44]. In such cases, the cells that are unable to undergo apoptosis encounter mutations that promote erratic cellular growth (such as a (t14;18) translocation leading to increased c-myelocytomatosis oncogene product (c-MYC) gene expression), and the impact is almost always going to be a rapid proliferation of the carcinogenic cells. This shows the great synergism between defective apoptosis and dysregulated cellular growth, for instance, the synergism between increased expression of Bcl-2 and c-MYC to give rise to follicular B-cell lymphoma [45]. Therefore, evasion of apoptosis is important for the continuous proliferation of malignant cells and tumor formation, and the ability to suppress apoptosis is considered one of the cancer hallmarks. As cancer grows, the instability of genes increases malignant cell variety. Chemotherapy and radiotherapy select tumor cells with a high threshold to initiate their intrinsic apoptosis pathway. This Darwinian selection procedure can cause the evolution of cancer-resistant treatment [46].

A significant technique promoting malignancy resistance to chemotherapy is apoptosis evasion. Some proteins within the apoptosis intrinsic pathway, like Bcl-2 and p53, appear to be distorted in resistant malignancies, so they aren't just promoting tumorigenesis but also contributing to MDR to cancer therapy. Deletions or inactivating mutations of Bax or Bak are uncommon, but many cancers that are resistant to treatment, including leukemia, gastric, and colon cancers, can demonstrate increased expression of pro-survival molecules such as Bcl-2 and Bcl-xL [47-49]. Actually, multiple papers have pointed out the fact that increased Bcl-2 expression correlates with worse malignancy prognosis, such as colorectal, prostate, bladder, colorectal, melanoma, lung, and breast cancers. On the other hand, Bcl-2 overexpression is related to chemoradiotherapy resistance [50]. The p53 gene can also undergo mutations, mostly missense mutations, which render apoptosis inactive, causing chemotherapy resistance [51].

Consistent with the upregulation of the anti-apoptotic proteins, Bcl-2 and p53, which are frequently suppressed, inhibitors of apoptosis proteins (IAPs) are usually overexpressed in multiple cancers conveying resistance to apoptosis and worsening disease [52,53]. IAP family members include survivin, inhibitors of apoptosis (c-IAP1 and c-IAP2), X-linked inhibitor of apoptosis (XIAP), BIR-repeat-containing ubiquitin-conjugating enzyme (BRUCE/Apollon), IAP-like protein 2 (ILP-2), neuronal apoptosis inhibitor protein (NAIP) and melanoma IAP (ML-IAP/Livin). IAPs family proteins contain a domain that acts as a ubiquitin ligase found in the ubiquitin-proteasome pathway catalyzing the degradation of target proteins. Furthermore, c-IAP1 and c-IAP2 are important organizers to control the nuclear factor kappa b (NF-κB) pathway and boost carcinogenesis by catalyzing NF-κB inducing kinase (NIK) breakdown [54]. Breast cancer patients encounter increased levels of survivin, XIAP, NAIP, c-IAP1, and c-IAP2 [55]. Inhibition of caspase-3 by increased XIAP levels has been reported in esophageal cancer [56]. High levels of c-IAP2 play an important role in the malignant progression of early pancreatic cancers [57]. Survivin inhibits apoptosis through uniting with XIAP to prevent XIAP breakdown by the ubiquitin-proteasome complex together with enhancing its inhibition of caspases, which in turn plays an important role in cancer chemoradiotherapy resistance and unfavorable outcome [58,59].

In one RCT, Bcl-2 was targeted by the Bcl-2 inhibitor AT-101 to boost radiotherapy efficiency in head and neck squamous cell carcinoma (HNSCC). Apoptosis was then detected by bisbenzimide stain for studying nuclear changes or by propidium iodide stain and flow cytometry for quantification of nuclear apoptosis. Adding AT-101 to radiotherapy was demonstrated depending on isobolographic analysis and through measuring the combination index (CI), which indicates the degree of interaction between AT-101 and radiotherapy. Patients who were assigned for the RCT were with locally advanced HNSCC and treated with cisplatin-based chemoradiotherapy, then were given oral dose-escalating AT-101 daily for two weeks in a schedule repeated every three weeks. The findings indicated that AT-101 potentiated radiotherapy-dependent apoptosis showing a CI of less than 1.0, which indicates synergism with radiotherapy. From that, we can conclude that AT-101 enhances radiation-dependent apoptosis in HNSCC in vitro, which supports adding At-101 to radiotherapy in malignancies with increased Bcl-2 expression [24].

Another RCT was conducted to demonstrate the effect of preoperative tyrosine kinase inhibitor vandetanib on markers of apoptosis and proliferation such as phosphorylated extracellular signal-regulated kinase (p-ERK) in rearranged during transfection (RET) positive breast cancers. Ten patients who had advanced breast cancer were given oral vandetanib 300 mg versus placebo over a period of two weeks before surgical removal of their cancers. Post-vandetanib treatment specimens were compared to pretreatment ones by immunohistochemistry for p-ERK. The findings indicated that there weren't any considerable changes in p-ERK activation in those who took vandetanib versus placebo. Therefore, the effects of vandetanib on RET-expressing tumors compared with placebo were not significant [23].

A quick summary of all included articles in this review is shown in Table 5 to demonstrate the aim and main findings of each article.

**Table 5:** A quick summary of all included articles in this review

Author of the publication	Aim of the study	Year of the study	Type of the study	Main findings
Neophytou et al. [16]	Reveal the major apoptosis pathways and illustrate how pro-apoptotic and anti-apoptotic proteins are modified in malignant cells to produce drug resistance.	2021	Review	Disruption of major apoptosis pathways can cause drug resistance. For example, disruption in B-cell lymphoma 2 (Bcl-2) levels and p53 inactivation have been seen in different types of multiple drug-resistant malignancies.
Mohammad et al. [17]	Provide a full picture of successful anti-cancer techniques that can overcome resistance to apoptosis to produce a better therapeutic outcome in patients with malignancy.	2015	Review	Apoptotic therapy needs a good choice of therapeutic techniques with a wide knowledge of determinants associated with therapy resistance, firstly by sorting the main reasons for apoptosis resistance and providing a list of prioritized targeted therapy.
Carneiro et al. [18]	Describe apoptosis pathways, signaling pathways that affect them, molecular targets, and clinical therapy.	2020	Review	Multiple pathways of inducing apoptosis are required in cancer cells, some more direct than others, mostly through the final common pathway that requires caspase-dependent proteolysis, membrane blebbing, and deoxyribonuclease (DNase)-dependent breakdown of chromosomal deoxyribonucleic acid (DNA).
Xu et al. [19]	Summarize the current understanding and knowledge of apoptosis and apoptotic bodies. Discuss apoptosis-related therapeutic applications.	2019	Review	There are different ways by which apoptosis can show itself, with multiple cells following different breakdown routes, eventually leading to the liberation of apoptotic bodies.
Strasser et al. [20]	Focus on the target of action of drugs that kill malignant cells by directly activating apoptosis machinery and synergizing with chemotherapy and targeted agents to provide better outcomes for cancer patients.	2020	Review	Clinically, the B-cell lymphoma 2 (Bcl-2) specific inhibitor venetoclax has been proven to be an excellent novel target for cancer treatment. Research on venetoclax is continuing with nearly 200 randomized controlled trials (RCTs) planned. These will eventually show the cancers that are more susceptible and the kind of resistance that may appear.
Singh et al. [21]	Provide recent insights into the dynamic relations between the B-cell lymphoma 2 (Bcl-2) proteins and how they control apoptotic cell death in cells to achieve new opportunities for therapeutic interventions.	2019	Review	Strong fundamental knowledge of Bcl-2 family protein function is crucial for choosing therapies, monitoring responses, and understanding mechanisms of drug resistance.
Yuan et al. [22]	Discuss the role of ubiquitination and deubiquitination in apoptosis and apoptotic cell clearance.	2022	Review	Ubiquitination is required for various cell functions and almost all aspects of growth and development. Multiple signaling pathways and genes are involved in ubiquitination.
Deng et al. [15]	Evaluate the relationship between the anti-apoptotic protein B-cell lymphoma 2 (Bcl-2) and neurological recovery in patients after traumatic brain injury (TBI).	2020	Systemic review	Improved connection between B-cell lymphoma 2 (Bcl-2) and apoptosis can help develop targeted therapies to decrease secondary neuronal loss. In neurons vulnerable to programmed cell death, increasing Bcl-2 levels produce a neuroprotective role and a field for a biomarker with diagnostic capability.
Spanheimer et al. [23]	Study the effect of taking vandetanib before surgery on proliferation and apoptosis markers in breast cancer.	2021	Randomized controlled trial	No statistically significant differences were shown with vandetanib compared to placebo. An unjustified claim was that treating with vandetanib will reduce phosphorylated extracellular signal-regulated kinase (p-ERK) and produce better effects in rearranged during transfection (RET)-expressing tumors.
Zerp et al. [24]	Evaluate combined effects of radiation and B-cell lymphoma 2 (Bcl-2) inhibitor AT-101 in head and neck squamous cell carcinoma (HNSCC).	2015	Randomized controlled trial	B-cell lymphoma 2 (Bcl-2) Inhibitor AT-101 leads to potentiation of radiotherapy-dependent apoptosis in head and neck squamous cell carcinoma in vitro, which further encourages the use of AT-101 in Bcl-2 expressing malignancies.

Limitations

The study has some limitations that should be considered. There are limited clinical trials with a special focus on pro-apoptotic therapy for cancer treatment. Access to free full text was also limiting the choice of articles. In addition, there is a lack of meta-analyses discussing apoptosis pathways and the relationship between cancer and evasion of apoptosis. Finally, there is a risk of information bias because of the widespread information gathered from different sources all over the world.

## Conclusions

This systematic review was carried out to provide ideas about apoptosis machinery and show the mechanism by which cancers can develop on top of cells incapable of undergoing apoptosis. In addition, it further assesses the efficacy of focusing on apoptosis as a potential target to overcome resistant cancers. From the results, we can conclude that apoptosis is a promising future potential target for cancer-related therapy due to the deep connection between cancer development and evasion of apoptosis. Still, we can also see that not all pro-apoptotic therapy can produce statistically significant results when added to conventional radio-chemotherapy to treat different cancers. Therefore, we recommend conducting a wide-range meta-analysis to provide a broad scale of information about the true efficacy of apoptotic therapy in cancer management.
